# Computational Efficiency of a Modular Reservoir Network for Image Recognition

**DOI:** 10.3389/fncom.2021.594337

**Published:** 2021-02-05

**Authors:** Yifan Dai, Hideaki Yamamoto, Masao Sakuraba, Shigeo Sato

**Affiliations:** Research Institute of Electrical Communication, Tohoku University, Sendai, Japan

**Keywords:** liquid state machine, reservoir computing, pattern recognition, robustness, Hough transform

## Abstract

Liquid state machine (LSM) is a type of recurrent spiking network with a strong relationship to neurophysiology and has achieved great success in time series processing. However, the computational cost of simulations and complex dynamics with time dependency limit the size and functionality of LSMs. This paper presents a large-scale bioinspired LSM with modular topology. We integrate the findings on the visual cortex that specifically designed input synapses can fit the activation of the real cortex and perform the Hough transform, a feature extraction algorithm used in digital image processing, without additional cost. We experimentally verify that such a combination can significantly improve the network functionality. The network performance is evaluated using the MNIST dataset where the image data are encoded into spiking series by Poisson coding. We show that the proposed structure can not only significantly reduce the computational complexity but also achieve higher performance compared to the structure of previous reported networks of a similar size. We also show that the proposed structure has better robustness against system damage than the small-world and random structures. We believe that the proposed computationally efficient method can greatly contribute to future applications of reservoir computing.

## Introduction

Recurrent neural networks (RNNs) have been successful in many fields including time sequence processing (Sak et al., 2014), pattern recognition (Graves et al., 2009), and biology (Maass et al., 2006; Lukosevicius and Jaeger, 2009). In a typical RNN, a network is initialized by randomly coupling the neurons, and their synaptic weights are updated by backpropagation as in feedforward networks. However, the major difference between RNNs and feedforward networks is that the connections in RNNs possess recurrent loops and thus create time-dependent dynamics. Higher degree of freedom gives RNNs the ability of generalization but also introduces design and training difficulties. The constraints of hardware and algorithms limit the applications of RNNs.

One practical paradigm to overcome the difficulties of RNNs is reservoir computing (RC) proposed by Maass et al. (2002a) and Jaeger (2001). The RC paradigm skips gradient-descent training in RNNs and uses a simple readout function to process the states of neurons. The network functionality is retained at a considerably reduced training cost (Maass et al., 2006). Therefore, RC performs well with limited resources. In addition, without backpropagation, spiking neuron models can be applied, leading to low power consumption of hardware (Soures, 2017; Zyarah et al., 2018). Such a model is generally referred to as liquid state machine (LSM), and previous works have confirmed that LSMs with specific connections are highly robust (Hazan and Manevitz, 2010, 2012) against both input noise and network noise, e.g., random attacks on a fraction of synapses. Efforts have been made to illustrate the relationship between LSMs and biological neuron networks (Lukosevicius and Jaeger, 2009) to find an explainable learning model based on biology.

Although LSMs have advantages, their practical application is still limited. This is primarily because of the lack of methods to scale up and the difficulty of parallelizing LSM simulations. This situation raises the following questions: (1) How can we improve the computational efficiency of reservoir networks? (2) How does the synaptic connection between the input and reservoir neurons influence the classification performance?

In this paper, regarding the first question, we consider a modular reservoir network and compare its performance with those of random, metric (Maass et al., 2002a,b), and small-world (SW) reservoirs. We show that the computational cost is minimized in modular networks, in which the simulation of the entire reservoir can be simplified using the divide-and-conquer method. Regarding the second question, we investigate the impact of the Hough transformation (Ballard, 1981), a model consistent with the mechanisms of information processing in the visual cortex (Kawakami and Okamoto, 1993), on the classification performance of image recognition tasks. We test the MNIST dataset by transforming the pixel values into sequences using Poisson coding. Owing to the randomness in the Poisson process, samples of the same class have very different firing times, which stresses the network generalization capabilities (Soures, 2017).

The outline of this paper is as follows: section Materials and Methods explains the methodology of the models. The experimental results together with noise analysis are illustrated in section Results. The discussion and conclusions are presented in sections Discussion and Conclusions.

## Materials and Methods

**Figure 1:**
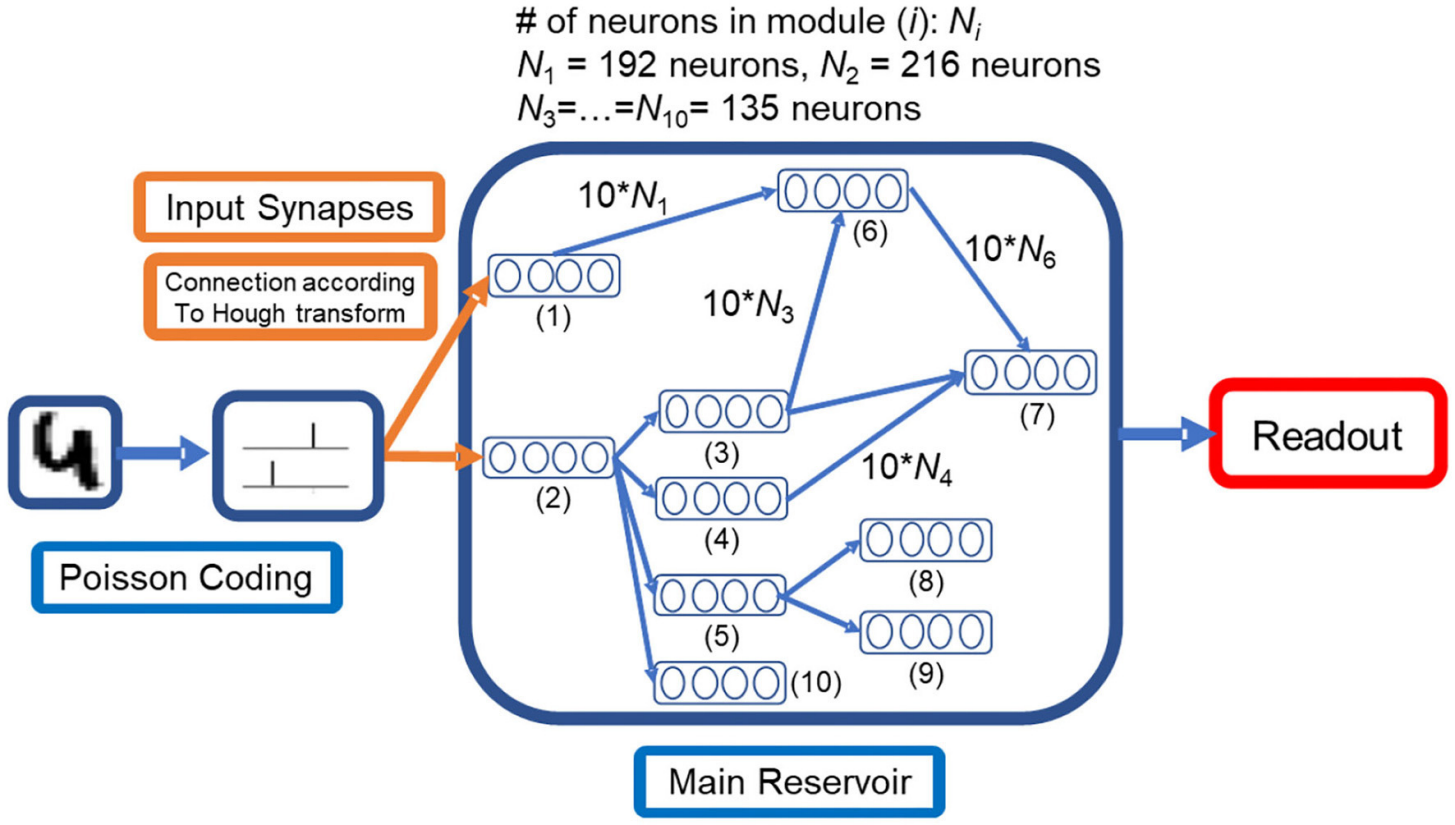
Proposed liquid state machine structure with a modular reservoir and specifically designed input synapses **Equations (1, 2)**, which perform the Hough transform with minor computational cost. The input image is first transformed into spikes by Poisson coding. Compared to feedforward structure, the states of the modules depend on multiple modules.

The LSM model consists of input, the spiking neuron reservoir, and readout. In our methodology, following the previous studies given in Kheradpisheh et al. (2018), we first preprocess the input using a difference of Gaussian (DoG) filter. DoG filter was used because it well approximates the center-surround properties of the retinal ganglion cells (Kheradpisheh et al., 2018). Next, the preprocessed sequences are encoded by Poisson coding and fed into the modular reservoir by Hough encoded synapses, as shown in Figure 1. The connection map of input synapses is predefined to make the firing rates of post-synaptic neurons consistent with the Hough transform of the input image (28 × 28-pixel MNIST images). The topology of the modular reservoir is described in subsection Reservoir Structure. Finally, a support vector machine (SVM) readout function with *C* = 1 is used to classify the reservoir states. Empirically, the linear kernel in SVM performs better than the Gaussian or polynomial kernel. From the experimental result presented in the following sections, all reservoirs perform better compared to the case where the input signal is classified using only the SVM without the reservoir. The basic simulation is performed in MATLAB.

### Preprocess

The input images are first preprocessed using the DoG filter. The size of the filter is 7, and the standard deviations of DoG are 1 and 2. After that, the float values are converted to temporal sequences by the Poisson coding method, where the maximum firing rate of each input neuron is 160 per second to keep the post-synaptic neuron firing neither too frequent nor too sparse. The total number of time steps per simulation is 1,600, with a time interval of 0.4 ms.

### Hough Transform

The Hough transform has a background in neurophysiology (Blasdel and Obermayer, 1994; Watts and Strogatz, 1998) and is an efficient method of image processing (Ballard, 1981). As illustrated in Figure 1, synapses between the input layer and the reservoir modules that receive direct inputs are designed to perform the Hough transforms.

Inspired by Kawakami and Okamoto (1993), we implement the Hough transform using a predefined map of input synapses using Equation (1) for lines and Equation (2) for circles:

(1) $$\begin{array}{l} W_{idx(\rho ,\;\theta ),\;idx(x,y)}=\delta_{\rho -x*\cos(\theta )-y*sin(\theta )} \end{array}$$

(2) $$\begin{array}{l} W_{idx(a,b,r),idx(x,y)}=\delta_{r^{2}-{(x-a)}^{2}-{(y-b)}^{2}} \end{array}$$

where *W*_*i,j*_ is the connection matrix between presynaptic (*j*) and post-synaptic (*i*) neurons, *idx(x,y)* is the index of the neuron with coordinates of *(x,y)* in the input layer, and δ*x* equals 1 if *x* is 1 and equals 0 otherwise. *(*ρ, θ*)* and *(a,b,r)* are the coordinates for post-synaptic modules, and *(x,y)* are for presynaptic modules. In this way, we decide whether or not to build a synapse between neurons *idx(x,y)* and *idx(a,b,r)* or *idx(*ρ, θ*)*. The Hough transforms for lines and circles consist of 16 × 12 and 6 × 6 × 6 neurons, respectively. Such a map integrates the synapses; thus, the firing frequency of post-synaptic neurons will consist of the Hough transform of the presynaptic layer. The response also agrees with the observation in the cortex well (Kawakami and Okamoto, 1993, 1995).

### Reservoir Structure

As shown in Figure 1, all modules in a modular reservoir are aligned to a directed acyclic graph. The graph is generated according to Algorithm 1 with 10 modules. The generation algorithm preserved the acyclic property between modules, which enables the division of computation load as discussed in section Computational Efficiency.

**Algorithm 1:** Random Directed Acyclic Graph Generation.

1. Fix the modules that are directly connected to input synapses
2. Label all modules (1, 2, …, *n*) according to the topologic order 3. For all non-input connections (from module *j* to module *i*, with *i* > *j*):
**if** rand < *p*: %% *p* (= 0.25) depends on the density of the graph
Connect module *j* and module *i*

Within each module, 9 × 15 neurons are connected according to the SW structure. We use the SW structure because it provides better connectivity with fewer synapses than a random network (Bohland and Minai, 2001). Moreover, the SW structure has high robustness to noise (Hazan and Manevitz, 2012). In terms of network dynamics, SW connectivity decreases the tendency for a network to be globally synchronized as compared to random networks (Netoff et al., 2004; Yamamoto et al., 2018b), and we expected that this leads to the maintenance of high dimensional dynamics as a reservoir. The SW network is generated by rewiring from a 2D regular lattice network where each neuron is connected to 8 neighbor neurons. The edges in the lattice are either preserved or rewired to another destination neuron with a probability of 0.35. Owing to the boundary effect, the average out-degree of neurons was 7.2. For neurons of different modules, the synapses form a directed acyclic graph. The connection map between two modules is aligned randomly. Each neuron in a presynaptic module is randomly connected to 10 neurons in a post-synaptic module in order to keep a moderate firing rate. Synaptic weights of connections between modules are kept constant.

This modular setting has previously been shown to increase the functional complexity of the network (Yamamoto et al., 2018a). We show experimentally that the modular reservoirs provide better performance over non-modular reservoirs of the same size. Moreover, the computation for the simulation of modular networks can be reduced using divide-and-conquer methods, as shown in Algorithm 2 and subsection Computational Efficiency.

**Algorithm 2:** Divide-and-Conquer Method for Modular Simulation.

1. Generate a directed acyclic graph and connect the modular reservoir where the modules of zero input degree are connected to the input layer
2. Add the input layer into a queue named *K* and label the depth 0. Add other modules into the set named *U*.
3. while (*U* != NULL):
4. *N*, depth = K.pop()
5. if *N*.neighbor_node != null:
6. parfor *I* in N.neighbor_node:
7. if *I* in *U*:
8. *I*.simulate()
9. *U*.drop(*I*)
10. *K*.add((*I*, depth+1))
11. %% Simulate each submodules separately according to the topology order, where modules of the same depth could be simulated in parallel. The results of the pre-modules are the input of the post-module.

### Neuron Model

The LSM model always consists of input, spiking neuron reservoir, and readout. The input is encoded by Poisson coding and the readout is one SVM with *C* equals 1. Empirically, the linear kernel in SVM performs better than the Gaussian or polynomial kernel. From the experimental result provided in the following subsections, all reservoirs perform better than in the SVM-only case where the input is directly fed into the SVM without the reservoir.

The neuron model of reservoir neurons is the leaky integrate-and-fire (LIF) model. The parameters of the LIF model used herein reference the work by Maass et al. (2002b) with a threshold *V*_th_ of 15 mV, a reset voltage *V*_reset_ of 13.5 mV, a membrane time constant τ_mem_ of 30 ms, *V*_rest_ of 13.5 mV, and refractory periods of 5 ms and 2 ms for excitatory (ex) and inhibitory (in) neurons, respectively. For the synaptic models, we use a delay of 1.5 ms for ex–ex (post–pre) and a delay of 0.8 ms otherwise. The strengths of synaptic weights are also referenced from Maass et al. (2002b) with slight modifications for reducing the mean firing rate: 20 for ex–ex, 45 for in–ex, and −17 otherwise. For each simulation, before presenting an input, a phase of 50 ms without any input is kept to allow all neurons decay to a resting value.

The activation of an LIF neuron is calculated as follows:

(3) $$\begin{array}{l} \tau_{mem}\frac{dV}{dt}=-[V(t)-V_{rest}]+RI(t) \end{array}$$

(4) $$\begin{array}{l} V(t)=V_{reset},ifV(t)=V_{th} \end{array}$$

Here, *R* and *V*_rest_ are constants and *I*(*t*) is the excitation current defined as *W*_in_×*S*_in_+*W*_res_×*S*_res_, where *W*_in_ and *W*_res_ are connection matrices of input–reservoir and reservoir–reservoir and *S*_in_ and *S*_res_ are spiking vectors of input neurons and reservoir neurons. After simulating for 1,600 time steps, the average spiking frequency of neurons in the reservoir is calculated and recorded as the features of the sample. Afterward, the collected features are fed into the readout SVM layer to perform the classification.

### Convolutional Neural Networks

To compare the performances of our LSM and a conventional network, we implement a trivial convolutional neural network (CNN) with two convolutional and subsampling layers and test it on the MNIST dataset. The kernels of the first and second layers have a 5 × 5 size with 6 and 12 channels, respectively. The reservoir network in the comparison group has 1,488 neurons with 10 modules. We simulate both networks on the same dataset until the models reach an accuracy of 90% and compare the convergence speed of the two networks.

## Results

We used the MNIST dataset to investigate the influence of the reservoir connectivity on the classification performance and computational load of LSMs. We compared the results of the proposed modular reservoir with those of other reservoirs, including SW, metric (Maass et al., 2002b), and random networks with comparable number of neurons and node degrees (Table 1). In addition, the LSM noise analysis is performed. The results show that the proposed modular structure has the highest robustness against both input noise and system noise.

**Table 1:** Summary of reservoir network configurations.

**Topology**	**Number of neurons**	**Parameters**
Random	1,500	Mean degree: 15 Connection probability: 0.01
Small-world	1,500	Mean degree: 8 Rewiring probability: 0.35
Metric	1,500 (Located on the integer points of a 15 × 10 × 10 lattice)	Mean degree: 9.44 Connection probability between nodes *a*-*b*: $P_{a,b}=C\cdot e^{\left[\frac{-D{\left(a,b\right)}^{2}}{\lambda^{2}}\right]}$, where *D*(*a,b*) is the distance between *a*-*b*, λ = 2, *C* = 0.3 (*ex-ex*), 0.2 (*ex-in*), 0.4 (*in-ex*), 0.1 (*in-in*).
Modular	1,488 (Module 1: 192, Module 2: 216, other Modules: 135)	Mean degree: 12.10 (See also section Reservoir Structure)

### Pattern Recognition

In this subsection, we present the results of 10-class classification tasks with the MNIST dataset, in which the input images are transformed into spike sequences. We set the test set to 200 randomly selected samples.

**Figure 2:**
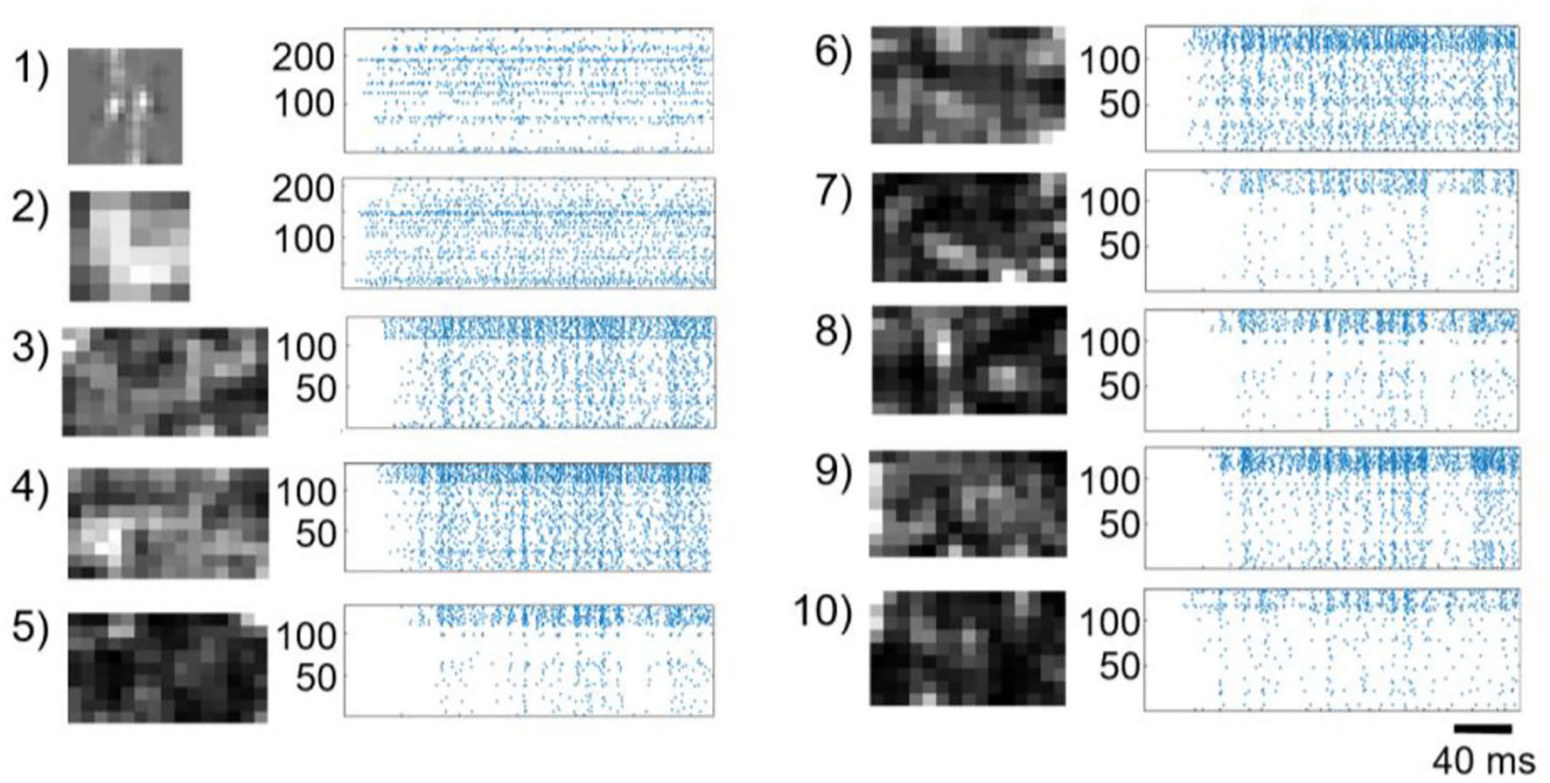
Firing rate and raster plot of each module in the proposed modular reservoir. The raster plot is an example of the network dynamics during learning of the symbol “4” preprocessed using a difference of Gaussian filter. The brightness of the pixels in the maps represents the firing rate of each neuron. As illustrated in Figure 1, the connections between modules are randomly generated to form an acyclic graph using **Algorithm 1**. The index of each graph indicates the index of the modules shown in Figure 1.

In Figure 2, we show the raster plots of neurons in different modules. We observe that each module exhibits different features that can be extracted by the SVM readout. For example, in the case of the input symbol “4,” there are two major vertical lines of zero degree in the polar coordinates, and consequently, there are two bright spots in the Hough-transformation layer of line detection with θ equals zero (module 1 in Figure 2). Similarly, in the second Hough-transformation layer, only one bright spot indicates that there is only one circular component with a fixed center in the symbol (module 2 in Figure 2). Within 10,000 of training samples, CNNs generally cannot converge, but the reservoir suffers only a slight loss. Therefore, the reservoir has the advantage of fast learning.

In order to validate the proposed structure, we compare it with the following cases: (1) an SVM classifier directly connected to the input (SVM-only); (2) a reservoir consisting of a metric network proposed by Maass et al. (2002b); (3) a common SW reservoir; and (4) a CNN with two convolutional and two subsampling layers. The metric network was constructed by connecting 1,500 neurons with the probability of $P_{a,b}=C\cdot e^{-\frac{{D\left(a,b\right)}^{2}}{\lambda^{2}}}$, where *C* and λ are constants and *D*(*a,b*) is the distance between nodes *a* and *b*. All parameters were adopted from Maass et al. (2002b) (Table 1). The SW reservoir was constructed using the same procedure described in subsection Reservoir Structure with 1,500 neurons.

**Figure 3:**
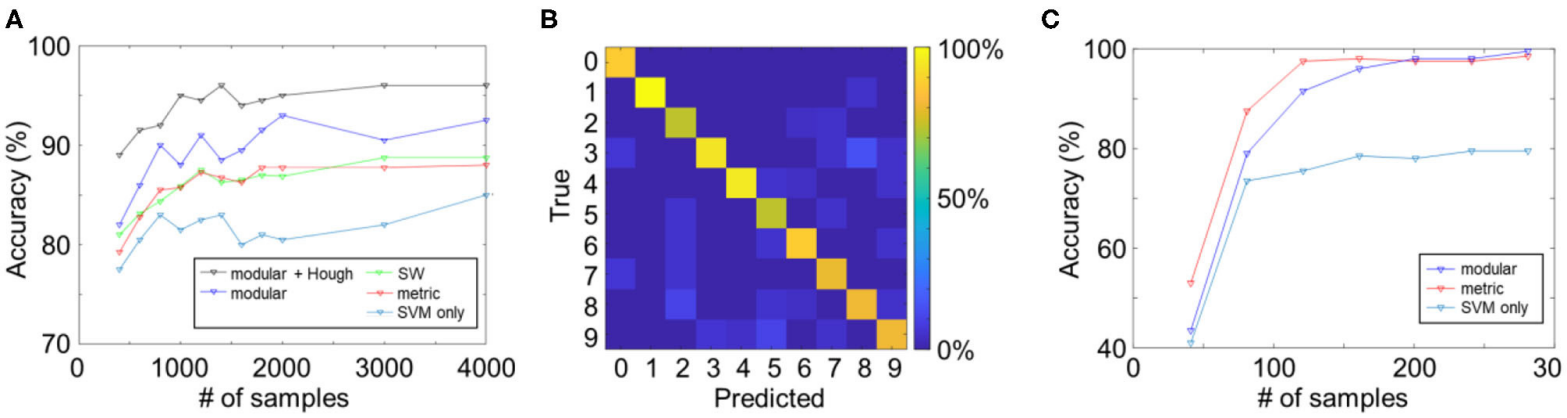
**(A)** Learning curves of reservoir computing models with different reservoir topologies. **(B)** Average confusion matrix for the modular + Hough reservoir network. **(C)** Learning curves of the reservoir computing models for the TI-46 classification task.

The learning curves of the reservoir networks are summarized in Figure 3A. All reservoir networks performed better than the SVM-only case, which verifies the effect of the reservoir on the computational performance. The highest score among the three topologies was achieved by the modular structure (black curve), possibly due to an increase in functional complexity in the reservoir network. Moreover, reservoir networks were found to converge significantly faster than CNNs (see subsection Comparison With the Convolutional Neural Network).

To better understand the classification performance of the reservoir networks, we created a confusion matrix with the predicted and true labels in columns and rows, respectively (Figure 3B). Two hundred images for each digit were fed into the network, and predicted labels were counted and presented as percentages. Interestingly, different digits with smaller distance were found to have higher percentage of being misclassified, e.g., “3” was classified as “8” in 14.3% of the trials. Contrarily, the misclassification was less frequent in digits with larger distance, and misclassification between “0” and “1,” e.g., was 0%.

The main enhancement of performance in the modular network comes from the Hough transform layer (compared the black and blue curves), which well supports our hypothesis that input synapses with specially designed connection patterns would largely improve the reservoir functionality. It is uncertain whether the Hough transform-based synapses are optimal. However, practically speaking, as the Hough transform is efficient in detecting the border of textures and is robust to image noise, the proposed connection pattern shows better performance over random input connections.

Considering the fact that reservoir networks are capable of efficiently learning sequential data, we also applied the proposed modular reservoir to the TI-46 dataset, a dataset of ten spoken digits (“0”–“9”). For this, the audio files were firstly preprocessed by the Lyon cochlear model into 78 channels using Auditory Toolbox in MATLAB (Slaney, 1998). Next, the first 100 ms of the preprocessed signals were clipped in order to eliminate background noise. Then, each channel of the signals was fed into input LIF neurons by input synapses. As the inputs in this test were audio signals, Hough transform-based connection was not applied, and each input neuron was randomly connected to 10 post-synaptic neurons. As shown in Figure 3C, the reservoir network with SVM readout function exceeded the SVM-only case, because of the separation property of the reservoir. Both the metric and modular reservoirs achieved a classification accuracy of over 95% with < 200 epochs. The difference between the two reservoir topologies, however, was insignificant in this task.

### Comparison With the Convolutional Neural Network

**Figure 4:**
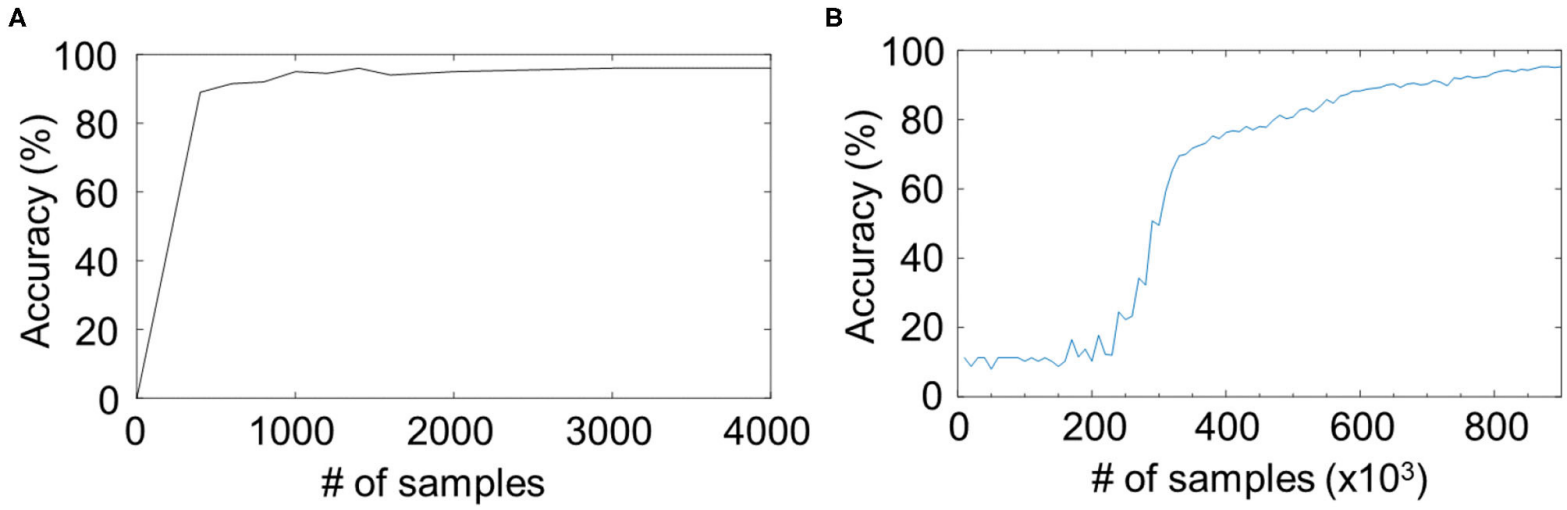
Comparison of the learning curves for **(A)** the proposed RC model and **(B)** a two-layer convolutional neural network (CNN). The same dataset as the “modular + Hough” in Figure 3A are shown in **(A)**. The RC model converges faster than the CNN.

The CNN is a more matured structure than the reservoir network, but in some fields without enough labeled data, the reservoir network may be more suitable because of the faster convergence speed. Comparison of the learning curves showed that the classification accuracy of reservoir network converged in the first 3,000 iterations (Figure 4A), whereas the CNN did not work until 300,000 iterations (Figure 4B). One reason is that reservoirs do not involve backpropagation, which can simplify the system and reduce the number of parameters from the number of connections to the number of neurons. Even using an unsupervised method, i.e., spike timing-dependent plasticity (STDP), in Kheradpisheh et al. (2018), a three-layer spiking system can converge within 5,000 iterations, which is still faster than backpropagation, because spiking sequences for a spiking network contain more information than float numbers for an artificial network. In absolute terms, state-of-the-art CNNs achieve >99.7% accuracy in MNIST classification tasks (Wan et al., 2013), which is higher than the accuracy of the presented reservoir networks. The two models, i.e., CNNs and reservoir computing, are not mutually exclusive and should thus be chosen depending of the conditions in applications, such as the amount of labeled data, network size, and the computational resource.

We note that the parameter space for the reservoir network is smaller or equivalent to that of the CNNs. For example, a simple CNN with two layers of 6 and 12 channels and kernels of size 5 × 5 has 1,950 parameters without counting the parameters in the dense linear map and normalization layers. In contrast, the number of parameters of the SVM readout function for the reservoir network in the experiments depends on the number of support vectors, which could be smaller than the number of parameters in dense linear map layers of CNN.

### Computational Efficiency

**Figure 5:**
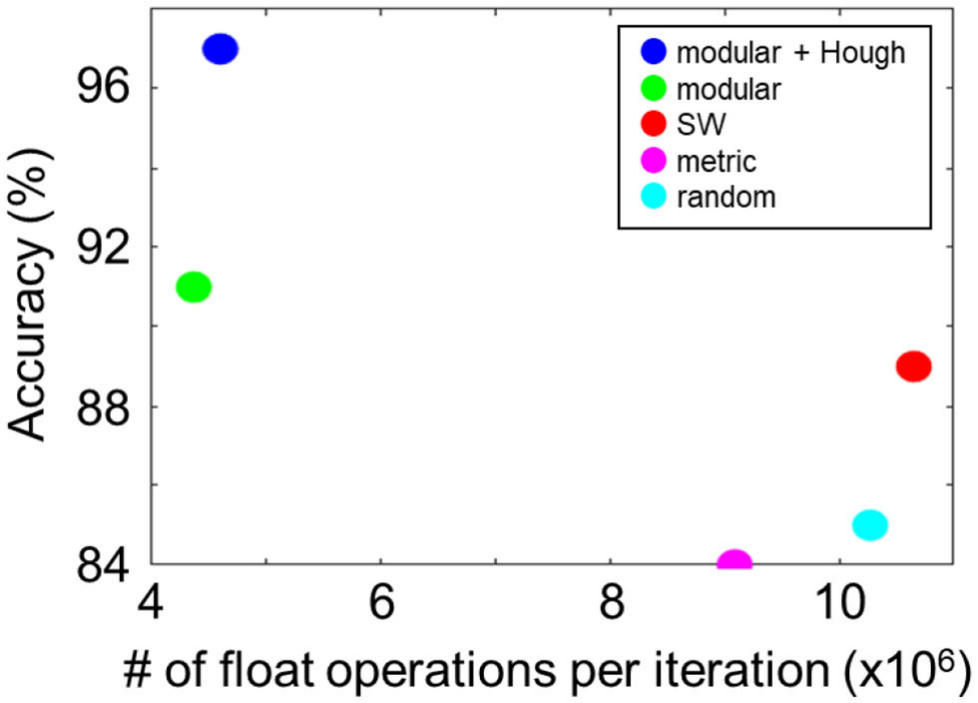
Computational cost (number of float operations per iteration) and performance of different network topologies. The proposed modular structure saves the cost significantly (blue and green).

Next, we compared the computational efficiencies of the LSMs with modular and non-modular reservoir networks by calculating the number of float operations per iteration. In Figure 5, the number of float operations per iteration is plotted for different settings. Among the non-modular networks, the metric structure exhibited the highest computational efficiency with 9.1 × 10^6^ FLOPS/iteration. The efficiency of the proposed modular structure was more than twice as high as that of the non-modular networks with 4.4 × 10^6^ FLOPS/iteration. Finally, we compared all configurations by evaluating the classification accuracy per million FLOPS, i.e., 21.0 (modular + Hough), 18.9 (modular), 9.0 (metric), 8.2 (SW), and 8.1 (random), quantitatively showing the advantage of the proposed LSM model.

The reason for the increased efficiency in the modular networks can be understood as follows. Suppose there are L modules with N neurons in each module. If the simulation is performed in the whole network, the matrix multiplexing cost is *O*((L*N)^*k*^) in time with *k* > 2. In contrast, in the case of the modular structure, we can simulate each module separately and reduce the cost for the entire network to *O*(L*N^k^) using the divide-and-conquer algorithm (Algorithm 2). Moreover, simulations for different modules can be performed in parallel according to the topological order. With greater number of modules, the improvement in the efficiency becomes more significant. For instance, on a personal computer with i7-core CPU and 8 GB memory, a 10-fold acceleration was achieved.

### Noise Analysis

One of the advantages of the RC paradigm is the robustness against noise. As described previously by Maass et al. (2002b), LSMs possess a separation property, which results in the reservoir robustness to input noise. However, for noise inside the system, which arises, e.g., by disabling some synapses, the robustness may substantially vary depending on the topology of the reservoir (Hazan and Manevitz, 2010). Especially for a random reservoir where the change of an arbitrary synapse uniformly influences every neuron in the network, disabling of some synapses causes significant deterioration. For a densely connected network, the situation worsens further.

**Figure 6:**
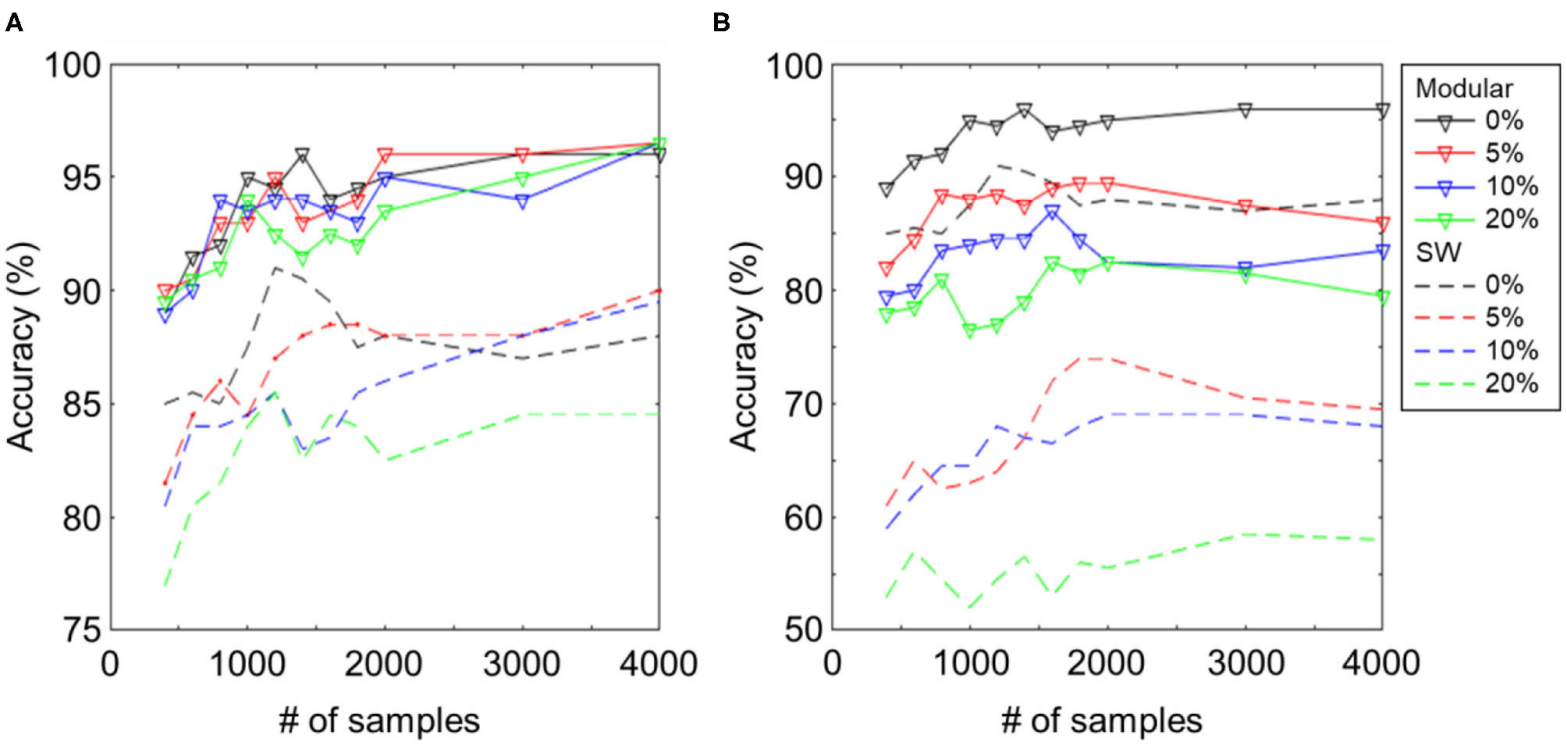
Classification accuracy for different levels of **(A)** input noise and **(B)** network noise (0/5/10/20% dead synapses). The solid lines denote the proposed modular structure and the dashed lines trivial SW reservoir. Both can resist input noise well as the reservoir computing paradigm suggests. However, the proposed structure shows better robustness to network noise than the trivial SW reservoir.

As shown in the previous work (Hazan and Manevitz, 2012), reservoir networks with hub nodes have higher robustness, as compared to random or SW networks, because the node degrees of a large fraction of nodes are low and hence hardly influence global dynamics. Here, we show that the proposed modular structure also has such a property by comparing it with the SW and metric (Maass et al., 2002b) networks. We compare the results under different levels of system noise, which was induced by disabling 0–20% of synapses. In Figure 6, we show the difference between the proposed structure and the SW reservoir of comparable sizes (Table 1). Both cases of input noise and system noise are displayed on left and right.

Figure 6A shows the learning curves for the SW (dashed) and modular (solid) reservoirs with the designated amount of white noise imposed on the input image. The strength of white noise is presented as a signal-to-noise ratio. The classification performance of both networks was robust against input noise, even under 20% of white noise, as the RC paradigm suggests. As shown in Figure 6B, a damage on 20% of synapses in the proposed modular structure decreased the classification accuracy by 18%, whereas for the SW structure, the accuracy decreased by 35%. Even in the case of 0.1% dead synapses, the SW and metric networks deteriorated substantially by 9 and 6%, respectively, whereas the deterioration was only 3% for the modular structure.

From the experimental results, we illustrate that owing to its decentralized property, the proposed modular structure can resist the damage to the network better than the simple one-module network topology, such as the SW connection. Our results indicate that for neuronal networks in the brain, which are known to be modular (van den Heuvel et al., 2016), the modular topology endows the network with high robustness to damage.

## Discussion

The RC has achieved superior results for various datasets including TI-46 (audio) (Verstraeten et al., 2005), DogCentric (video activity recognition) (Soures and Kudithipudi, 2019), etc. Compared to traditional RNNs, RC saves the resource for backpropagation. However, because of the difficulties in parallelization, the simulation of spiking networks is still time-consuming depending on the precision of models. Therefore, hardware-based parallelization speedup could be a promising direction for LSMs.

From the experiments, we showed that the proposed modular reservoir network significantly improves the performance and robustness compared to non-modular structures, because the changes in modules do not have global influence. Moreover, the modular method substantially reduces the computational cost, as shown in Figure 5, which enables further expansion of the network size. The results also showed that the classification accuracy in modular networks was higher as compared to metric and SW networks. Although the in-depth understanding of the core parameters that led to this improvement in the modular topology requires further systematic experiments investigating the parameter space for each network configurations, we speculate that separate modules functioning as independent classifiers reduced the variation, or overfitting, similar to the mechanics in a random forest.

The use of the Hough transform in the connection of input synapses is very efficient for image pattern recognition, as shown in Figure 3A. Although it is uncertain whether the Hough transform-based connection is the optimum setting for synapses, our results showed that it improves the performance, as compared to the random connection. Further optimization could be performed using STDP or pruning.

The proposed setting for the LSM is biologically plausible. First, the use of Hough transform-based input synapses agrees well with the model of motion detection in the visual cortex (Kawakami and Okamoto, 1993, 1995). The responses of neurons in the visual cortex are known to match the activities of the Hough transform layers of spiking neurons. In addition, the modular architecture is an evolutionarily conserved connectivity pattern found in the nervous system of animals (van den Heuvel et al., 2016). Moreover, the robustness to noise of the proposed network is consistent with the fact that the biological neuronal system resists damage well.

Recently, Cramer et al. (2020) showed that reservoir networks of spiking neurons require different operating points depending on task complexity. The optimal topology within each module thus may vary depending on the nature of the tasks, but the advantage of modular reservoirs being robust and computationally inexpensive should remain consistent irrespective of the tasks. We also note that, in the current work, fixed values of connection strengths and mean degree were used for the excitatory and inhibitory connections and that the connections between modules were randomly formed. Further optimization of the parameters, as well as the intermodular coupling scheme, should further improve the performance of the modular reservoirs.

The model explored in this work can be viewed as a type of deep spiking networks. Previously, Kheradpisheh et al. (2018) reported on deep spiking CNNs, in which weights are optimized using STDP, and achieved superior results on various datasets. The simplified STDP used in that work can be a potential method to prune synapses in the modular reservoir, given that the values of learned synapses converged to the upper and lower limits. The deep feedforward LSM explored in Soures (2017) and Soures and Kudithipudi (2019) is similar to the proposed network in terms of network topology. We think that by introducing acyclic directed modules in feedforward LSMs, the network functionality can be further extended and its applications can be generalized like in residual networks or graph networks.

In comparison with deep CNNs, RC still has a long way to practical applications. Nevertheless, the RC paradigm could replace CNNs in fields where labeled training sets are not sufficiently large. The RC ability to extract time-dependent features is also important. We think that the proposed computationally efficient method can greatly expand the future application of RC.

## Conclusions

We showed experimentally that reservoir networks with modular topology and Hough transform-based input synapses can not only efficiently scale up the performance of pattern recognition in LSMs, but also improve the system robustness and resistance against system damage. It is obvious that different topologies influence the network functionality (Hazan and Manevitz, 2010, 2012), which indicates the existence of the most efficient system for a given problem. We believe that the current network topology can be optimized further, e.g., using unsupervised methods, such as STDP. Several attempts have been made to implement STDP in reservoir networks (Indiveri, 2002; Li and Jin, 2016). However, the performance of those methods after a scaleup still needs to be confirmed (Nessler et al., 2013; Diehl and Cook, 2015). The visualization and explainability, which are issues in deep neural networks, need to be considered in reservoir networks as well. Our future work will focus on the application of unsupervised techniques, such as various STDP rules, to improve the functionality and interpretability of large-scale reservoir networks. In addition, we will further explore the relationship to neurophysiology to achieve biologically plausible models with engineering efficiency.

## Data Availability Statement

The raw data supporting the conclusions of this article will be made available by the authors, without undue reservation.

## Author Contributions

YD and SS designed the research with inputs from MS. YD coded the simulation and analyzed the results. YD and HY wrote the paper. All authors reviewed and edited the manuscript.

## Conflict of Interest

The authors declare that the research was conducted in the absence of any commercial or financial relationships that could be construed as a potential conflict of interest.
